# Understanding Glycogen Storage Disease Type IX: A Systematic Review with Clinical Focus—Why It Is Not Benign and Requires Vigilance

**DOI:** 10.3390/genes16050584

**Published:** 2025-05-15

**Authors:** Egidio Candela, Giulia Montanari, Andrea Zanaroli, Federico Baronio, Rita Ortolano, Giacomo Biasucci, Marcello Lanari

**Affiliations:** 1 Pediatric Unit, IRCCS Azienda Ospedaliero-Universitaria di Bologna, 40138 Bologna, Italy; egidio.candela2@unibo.it (E.C.); rita.ortolano@aosp.bo.it (R.O.); marcello.lanari@unibo.it (M.L.); 2 Department of Medical and Surgical Sciences, Alma Mater Studiorum, University of Bologna, via Massarenti 11, 40138 Bologna, Italy; 3 Specialty School of Pediatrics, Alma Mater Studiorum, University of Bologna, 40126 Bologna, Italy; giulia.montanari22@studio.unibo.it (G.M.);; 4 Pediatrics and Neonatology Unit, Guglielmo da Saliceto Hospital, 29121 Piacenza, Italy; giacomo.biasucci@unipr.it; 5 Department of Medicine and Surgery, University of Parma, 43126 Parma, Italy

**Keywords:** glycogen storage disease type IX, PHKA1, PHKA2, PHKB, PHKG2, PHK, expanded newborn screening, GSD, GSD IX, metabolic disease, Glycosade, Maizena, uncooked corn starch, high-protein intake, next-generation sequencing

## Abstract

Background/Objectives: Glycogen storage disease type IX (GSD IX) is a group of inherited metabolic disorders caused by phosphorylase kinase deficiency affecting the liver or muscle. Despite being relatively common among GSDs, GSD IX remains underexplored. Methods: A systematic review of GSD IX was conducted per PRISMA guidelines using SCOPUS and PubMed, registered with PROSPERO. Inclusion focused on human clinical studies published up to 31 December 2024. Results: A total of 400 patients with GSD IX were analyzed: 274 IXa (mean age at diagnosis 5.1 years), 72 IXc (mean age at diagnosis 4.9 years), 39 IXb (mean age at diagnosis 4.2 years), and 15 IXd (mean age at diagnosis 44.9 years). Hepatomegaly was commonly reported in types IXa, IXb, and especially IXc (91.7%), but was rare in IXd. Elevated transaminases were frequently observed in types IXa, IXb, and particularly IXc, while uncommon in IXd. Fasting hypoglycemia was occasionally observed in types IXa and IXb, more frequently in IXc (52.7%), and was not reported in IXd. Growth delay or short stature was observed in a substantial proportion of patients with types IXa (43.8%), IXb, and IXc, but was rare in IXd. Muscle involvement was prominent in IXd, with all patients showing elevated CPK (mean 1011 U/L). Neurological involvement was infrequently reported in types IXa and IXc. Conclusions: This systematic review includes the most extensive clinical case history of GSD IX described in the literature. The clinical spectrum of GSD IX varies widely among subtypes, with IXc being the most aggressive. While liver forms are generally present in early childhood, muscle-type IXd shows delayed onset and milder symptoms, often leading to diagnostic delays. For diagnosis, it is essential not to underestimate key clinical features such as hepatic involvement and hypoglycemia in a child under 5 years of age. Other manifestations, including the as-yet unexplored systemic involvement of bone and kidney, remain insufficiently understood and require further investigation. Next-generation sequencing has improved diagnostic precision over traditional biopsy. Dietary management, including uncooked cornstarch, Glycosade^®^, and high-protein intake, remains the cornerstone of treatment. However, there is a paucity of well-designed, evidence-based studies to determine the most effective therapeutic approach. Despite its historically perceived benign course, the broad phenotypic variability of GSD IX, including progressive liver involvement and potential neurological complications, highlights its substantial clinical relevance and underscores the need for accurate diagnostic classification and long-term multidisciplinary follow-up.

## 1. Introduction

Glycogen storage diseases (GSDs) comprise a group of inherited metabolic conditions arising from enzymatic defects that impair glycogen synthesis or degradation [1].

Among these, GSD type IX (GSD-IX, OMIM 306000) is due to impaired activity of phosphorylase kinase (PhK), a regulatory enzyme involved in glycogen catabolism [2].

PhK is a heterotetramer composed of four distinct subunits (α, β, γ, and δ), each exhibiting tissue-specific isoforms encoded by different genes [3,4].

Liver-specific isoforms of the α, β, and γ subunits are encoded by the PHKA2, PHKB, and PHKG2 genes, respectively, and their dysfunction leads to GSD IX subtypes IXa, IXb, and IXc.

In contrast, the PHKA1 gene encodes for a muscle-specific subunit and GSD IX subtype IXd, with very few cases described in the literature.

PhK is present in multiple tissues, including the brain [5]. Nevertheless, the exact pathways through which PhK deficiency influences physical and mental development have yet to be elucidated.

PhK deficiency is classified into two primary types: liver PhK deficiency and muscle PhK deficiency. Liver PhK deficiency typically presents in early childhood and is characterized by hepatomegaly, growth restriction, and, in many cases, fasting ketosis and hypoglycemia; however, the latter is not always observed [6]. While it was previously believed that the clinical symptoms and biochemical abnormalities associated with liver PhK deficiency improve with age, emerging evidence suggests that affected individuals require long-term monitoring due to the risk of complications such as liver fibrosis and cirrhosis. Muscle PhK deficiency, which is significantly rarer, predominantly affects muscle function and is associated with symptoms such as exercise intolerance, myalgia, muscle cramps, episodes of myoglobinuria, and, in some cases, progressive muscle weakness [7]. These distinctions underscore the importance of meticulous clinical management in preventing potential long-term complications.

The prevalence of GSD type IX is approximately 1 in 100,000, representing approximately 25% of all GSD, with GSD IXa accounting for about 75% of all GSD IX cases [8].

Although GSD IX is one of the most commonly diagnosed forms of glycogen storage disease, it remains relatively underexplored, with significant gaps in our understanding of its natural history, molecular mechanisms, and optimal therapeutic approaches. This systematic review aims to synthesize existing knowledge on GSD IX. The primary focus is to describe the clinic, provide an overview of the genetics, diagnosis, and treatment, and offer our speculations while addressing key areas that warrant further investigation.

## 2. Materials and Methods

### 2.1. Search Strategy

A systematic review was carried out in alignment with the Preferred Reporting Items for Systematic Reviews and Meta-Analyses (PRISMA) guidelines [9], and the protocol was prospectively registered on PROSPERO (registration number: CRD42024622502). Supplementary Table S1 provides an overview.

The review specifically focused on glycogen storage disease type IX.

A comprehensive search of SCOPUS and PubMed will be performed utilizing both individual keywords and MeSH terms. To enhance article retrieval, search terms will be strategically combined using Boolean operators (“AND”) and refined through database-specific filters.

A purposely defined search string will be performed for PubMed: (“Glycogen Storage Disease Type Ix” [Mesh]) and SCOPUS: TITLE-ABS-KEY (Glycogen AND Storage AND Disease AND Type AND ix). Further literature searches were conducted using the following terms: “PHKA1”, “PHKA2”, “PHKB”, “PHKG2”, “GSD AND IXa”, “GSD AND IXb”, “GSD AND IXc”, and ‘’GSD AND IXd’’ to achieve maximum coverage of reported cases.

The search will include all articles published up to 31 December 2024.

### 2.2. Study Selection

The inclusion and exclusion criteria were established prior to the initiation of the review process.

Studies were considered eligible if they met the following criteria: (1) inclusion of human subjects diagnosed with glycogen storage disease type IX; (2) provision of clinical or laboratory outcome data; and (3) publication in the English language. Exclusion criteria included: (1) articles classified as reviews, letters, or editorials; and (2) studies lacking sufficient individual clinical data.

The results were reported as laboratory parameters with respective units, reference ranges, and mean values. Clinical features were described individually and incidence rates were reported.

**Figure 1:**
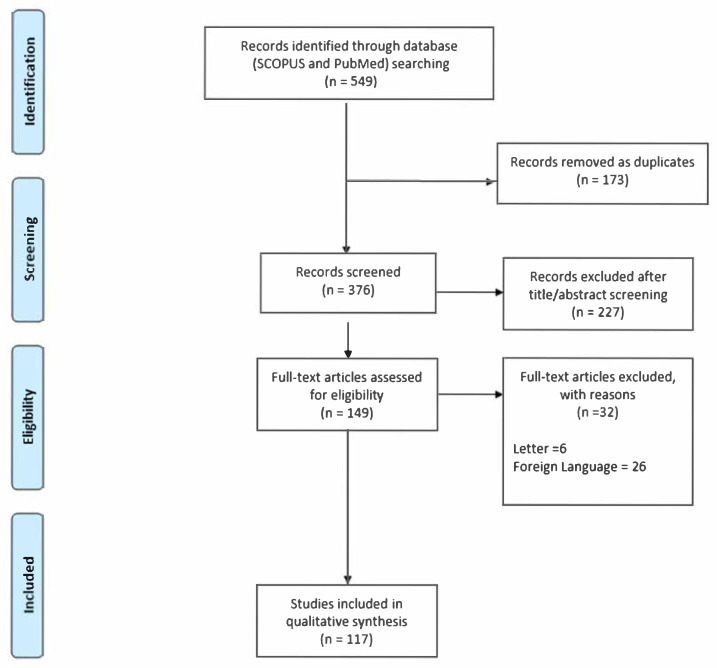
Flow diagram according to PRISMA guidelines.

Following the removal of duplicates, an initial screening of titles and abstracts was conducted. Full-text articles were then retrieved for studies deemed potentially relevant and assessed for eligibility and data extraction in accordance with PRISMA guidelines (as illustrated in Figure 1).

### 2.3. Data Extraction

Data from the included studies were then extracted, organized, and analyzed in Microsoft Excel 2019 (Microsoft Corp, Redmond, WA, USA). Three independent authors (EC, AZ, and GM) extracted data. Risk of bias in the included studies was assessed qualitatively by three independent reviewers (EC, AZ, and GM), who evaluated each study based on study design, reporting completeness, and clarity of clinical data. The reviewers worked independently and disagreements were resolved through discussion with a fourth reviewer (FB). No standardized risk of bias assessment tools or automation tools were used in this process.

## 3. Results

### 3.1. Genetics and Pathogenesis

GSD IX results from a deficiency in the PhK enzyme, leading to impaired glycogen breakdown. Phosphorylase kinase plays a critical role in hepatic glucose metabolism by activating liver glycogen phosphorylase (PYGL) through reversible phosphorylation at serine residue 15, converting phosphorylase b to its active form, phosphorylase a, which then facilitates the breakdown of glycogen into glucose 1-phosphate (G1P) monomers [10]. In the interprandial state, PhK, activated by glucagon, facilitates glycogenolysis by stimulating glycogen phosphorylase to release glucose from glycogen, thereby maintaining normal blood glucose levels. Defects in PYGL or PhK disrupt this process, leading to impaired glycogen breakdown and resulting in glycogen accumulation. This enzymatic defect leads to clinical manifestations such as hepatomegaly, ketotic hypoglycemia, and impaired growth in individuals with GSD [11].

PhK is a holoenzyme with a molecular mass of approximately 1.3 MDa, consisting of a hexadecameric structure formed by four repeats of each of its four subunits: α, β, γ, and δ. Among them, the γ subunit is solely responsible for catalytic activity, whereas the remaining subunits serve regulatory roles [12]. Pathogenic variants in the following genes lead to distinct forms of PhK deficiency:

- **PHKA1**, which encodes the α subunit, is implicated in a rare form of phosphorylase kinase deficiency restricted to muscle tissue.
- **PHKA2**, also encoding the α subunit, is linked to the most prevalent form, characterized by hepatic PhK deficiency.
- **PHKB,** coding for the β subunit, results in a form of the disease involving both hepatic and muscular tissues.
- **PHKG2**, responsible for encoding the γ subunit, underlies liver-specific PhK deficiency.

The δ subunit of PhK, known as calmodulin, is encoded by three separate genes—CALM1, CALM2, and CALM3—yet, to date, no mutations in these genes or in PHKG1 have been associated with phosphorylase kinase deficiency [13]. Additionally, subtype IXa is further divided into XLG I and XLG II, two clinically similar entities that differ primarily in the ability to detect enzyme deficiency in erythrocytes [14].

PhK deficiency affecting the liver due to PHKA2 mutations and the muscle-specific form caused by PHKA1 mutations are both inherited in an X-linked manner [15]. Conversely, PhK deficiency involving both liver and muscle, resulting from PHKB mutations, as well as the hepatic form due to PHKG2 mutations, follows an autosomal recessive pattern of inheritance (Table 1) [13].

Attempts to correlate genotype and phenotype have been made on several occasions.

Different clinical manifestations have also been found in the same mutation, such as patients carrying the *p.P1205L* mutation exhibited remarkable variability in clinical presentation [16]. Some individuals presented with mild hepatomegaly accompanied by atopic dermatitis and recurrent diarrhea. In contrast, others displayed features resembling GSD Ia, including severe hepatomegaly, elevated transaminases, short stature, and recurrent hypoglycemia, necessitating intensive dietary management.

A recent study by Geramizadeh et al. [17] provides a comprehensive review and explores the correlation between genotype and phenotype.

### 3.2. Clinical Presentation

The clinical and laboratory characteristics of the patients are shown in Table 2.

The articles from which the patients are drawn are given in Supplementary Table S2 [2,3,7,8,14,15,16,18,19,20,21,22,23,24,25,26,27,28,29,30,31,32,33,34,35,36,37,38,39,40,41,42,43,44,45,46,47,48,49,50,51,52,53,54,55,56,57,58,59,60,61,62,63,64,65,66,67,68,69,70,71,72,73,74,75,76,77,78,79,80,81,82,83,84,85,86,87,88,89,90,91,92,93,94,95,96,97,98,99,100,101,102,103,104,105,106,107,108,109,110,111,112,113,114,115,116,117,118,119,120,121,122,123,124,125,126,127,128]. No formal assessment of reporting bias was performed due to the descriptive nature of the synthesis and variability in data reporting across included studies. The certainty of the evidence for each outcome was not formally assessed. Given the descriptive nature of the synthesis and the predominance of case reports and case series, a formal evaluation of confidence in the body of evidence (e.g., using GRADE) was not applicable.

#### 3.2.1. GSD IXa (PHKA2 Related)

A total of 274 patients with liver-type GSD IXa were identified in the literature.

GSD IXa is the most common form of this group of glycogen storage diseases, accounting for approximately 68% of total GSD IX cases.

In 1994, Hendrickx et al. [14] first described a new type of X-linked liver glycogenosis with primary symptoms of liver enlargement and growth retardation. Key clinical manifestations of GSD IXa typically include liver involvement, growth impairment, and episodes of hypoglycemia.

The two main signs concern hepatic involvement and can be distinguished between hepatomegaly, sometimes reported as abdominal distension, which is described in 74.8% of patients, and transaminase elevation, described in 63.9% of patients.

Growth delay or short stature was reported in 43.4% of GSD IXa, not always with differentiation between the two conditions. In one neonatal case, infrequent lactic acidosis occurs in a patient carrying other mutations besides PKHA2 [45].

Neurological symptomatology has been described in several cases: one study reported psychomotor retardation, seizure, and autism signs in GSD IXa [50]. Another patient with glycogenosis IXa had simultaneous autism, increased transaminases, and short stature [46]. A report described two adult brothers who presented with neonatal hepatosplenomegaly, followed by later onset of hearing loss, cognitive impairment, and cerebellar involvement [59].

Anecdotal doll-face has been described only in one patient [112] in this form of glycogenosis.

#### 3.2.2. GSD IXb (PHKB Related)

A total of 39 cases of GSD IXb were collected from the literature.

Hepatic manifestations are similar to those of IXa, including hepatomegaly (79.5%), transaminase elevation (53.8% of patients), and fasting hypoglycemia (25.6%).

The clinical and laboratory characteristics are presented in Table 2.

Neonatal clinical manifestation is infrequent, although it was described in a 2022 case report in a preterm infant (33 weeks gestational age at birth) brought to the emergency room at 27 days of life with hypoglycemic coma [31]. The subsequent diagnosis of GSD IXb was made through whole-exome sequencing.

The doll face was described in this form of glycogenosis in a single patient in 1997 [113].

#### 3.2.3. GSD IXc (PHKG2 Related)

A total of 72 patients diagnosed with liver type GSD IXc were retrieved from the literature, with clinical and laboratory characteristics presented in Table 2**.**

This form of glycogenosis can mimic both hepatic glycogenosis types I and III, being characterized by the early onset in early childhood of hepatomegaly, altered lipid balance, and growth retardation [52,127].

Severe hepatomegaly often presents earlier than in other subtypes, also in the neonatal period [30].

Progression to fibrosis or cirrhosis is uncommon. However, specific genotypes associated with an absolute enzyme deficiency may be determinants and a predisposing factor for cirrhosis [24,118].

A recent case report [49] reports the story of a woman with a late diagnosis of GSD IXc (at 68 years of age) who presented with the clinical picture of hypoglycemia, hepatomegaly, and short stature and died of cirrhosis and recurrent multiple hepatocellular adenomas at the age of 69 years and 11 months.

#### 3.2.4. GSD IXd (PHKA1 Related)

A total of 15 cases of GSD IXd were collected from the literature.

Consistent with the X-linked transmission of the disease, almost all the patients reported were male (93.3%), with only one case described of a female patient, who presented with camptocormia and late-onset myopathy and was only diagnosed following the identification of the disease in her brother [51].

The mean age at clinical onset was 35.9 years (2.2–72 years). It is known that patients may remain asymptomatic for long periods and, in many cases, only show symptoms following intense physical exertion [90]. According to some authors [97], this clinical course could be explained by the fact that GSD IXd is a mild metabolic myopathy, characterized by reduced muscle glycogen catabolism and impaired lactate production during dynamic exercise, but with only a marginal impact on oxidative capacity. Other authors hypothesize that the moderate-to-light muscle symptoms can be attributed to the activation, under intense exercise conditions, of an alternative glycogenolysis pathway stimulated by AMP and inorganic phosphate (Pi), activators of myophosphorylase [90].

The symptoms are, therefore, subtle and nuanced, affecting the muscular system with myalgia, cramps, exercise intolerance, muscle weakness, and—in rare cases—post-exercise pigmenturia. The distribution of muscle involvement may be either proximal or distal. The average age at diagnosis was 44.9 years (range: 16–78 years), reflecting the diagnostic difficulty and frequent underestimation of the condition. For this reason, it was suggested to include GSD IXd in the differential diagnosis of exercise intolerance disorders, with a recommendation to have patients undergo provocative tests such as the ischemic forearm exercise test and the maximal multistage 20-m shuttle run test [40].

In cases where a biopsy was performed, this was a muscle biopsy, consistent with the predominant symptomatology. All patients analyzed had elevated Creatine Phosphokinase (CPK) values, averaging 1011 U/L. Histological findings showed glycogen accumulation and reduced or absent residual enzyme activity of PhK. In only one case [124], both liver and muscle biopsies were performed: while liver phosphorylase b kinase activity was regular, muscle phosphorylase b kinase activity was almost completely absent (0.5% of the minimal control value).

No cases of fasting hypoglycemia, hypertriglyceridemia, hypercholesterolemia, or cardiomyopathy were reported.

In contrast, neurological involvement with cognitive impairment was described in two patients. In one patient, who presented with short stature, cognitive impairment, obesity, and progressive myopathy, a novel frameshift mutation (c.2594delA) in the PHKA1 gene was found [51]. In the other case, published in 2010 [94], the patient showed cognitive impairment but no overt myopathy, with a de novo mutation (c.del1394T). The authors hypothesized a possible central nervous system involvement in GSD IXd, associated with encephalic glycogen accumulation, similar to what has been observed in some cases of Pompe disease.

Lastly, the literature reports a total of two cases of liver-type GSD IXd: one case of a patient with hepatomegaly, hypertransaminasemia, thrombocytopenia, and muscle involvement [50], and the other in a patient with a very rare co-occurrence diagnosis of GSD IXd and GSD II [7].

### 3.3. Overview of Diagnosis

GSD type IX is among the most challenging GSD s to diagnose [129]. In a study where 12 physicians with expertise in hepatic GSDs reviewed 45 standardized clinical cases, the overall diagnostic accuracy was 47%. GSD Ia and Ib were the most accurately identified, while no expert correctly diagnosed GSD IXc. The diagnostic accuracy for GSD IX cases was only 13%, significantly lower than the overall average of 47% [28].

Historically, the diagnosis of glycogen storage disease type IX relied on liver biopsy and the assessment of hepatic glycogen phosphorylase activity in frozen liver tissue, as well as in leukocytes and erythrocytes. However, the complexity of phosphorylase kinase, which contributes to the marked phenotypic variability of the disease, has made diagnosis particularly challenging [2]. The interpretation of PhK activity measurements in the liver, erythrocytes, leukocytes, and muscle tissue is further complicated by the potential for both false-positive and false-negative results [6].

Since PhK activity analysis fails to rule out PhK deficiency and is also an invasive test, it currently seems more reliable to perform a DNA test, most often a next-generation sequencing (NGS) panel, to confirm GSD IX [6].

The use of liver biopsy, burdened by both greater invasiveness and lower specificity, with the risk of damaging the liver’s glycogen stores, remains prerogative today only in developing countries. Indeed, one example is that of India [37], where in the first and largest case series recently described, liver biopsy was still performed in the majority (84%) for diagnosis.

Another concrete effect of the spread of various NGS genetic panels as a preferable diagnostic tool [81,130], in addition to lower cost and easier access, is the reduction of diagnosis time in GSD IX patients, as demonstrated in a study conducted by Aslı İnci et al. [36].

An interesting Korean pilot study [131] performed a Rapid Targeted Sequencing trial, designed by a multidisciplinary team, on dry blood samples restricted exclusively to infants with suspected actionable genetic diseases, including the PHKA2 gene.

It is essential to observe how, while most clinical laboratories provide gene sequencing panels, an important focus is the supplement of NGS with Sanger sequencing to ensure comprehensive coverage of poorly sequenced exonic regions [6].

Moreover, if available, the use of whole-exome sequencing (WES) enhances diagnostic yield and facilitates precision management in pediatric cases of unknown etiology with a well-founded clinical suspicion [23]. For families with a GSD proband, pedigree analysis and genetic testing are highly recommended [58].

Regarding the appropriateness of including this class of glycogenosis in newborn screening, interesting work has shown that the value of biotinidase enzyme activity in these patients is high, with a sensitivity of 100% for patients with GSD Ia, GSD I non-a, and GSD VI, 62% for GSD III, and 77% for GSD IX [99].

### 3.4. Overview of Management and Treatment

Although the severity varies among the different liver forms of glycogen storage disease, hypoglycemia remains the most feared clinical complication and, therefore, the first therapeutic target. The primary goals of management and treatment are to maintain stable blood glucose levels, correct secondary metabolic imbalances, and minimize long-term complications, all while ensuring a good quality of life.

To achieve these goals, metabolic control is monitored by combinations of clinical and biochemical markers, even if, over time, continuous glucose monitoring (CGM) systems from the diabetes experience have proved very useful and have become increasingly popular, quickly becoming a widespread aid for patients and clinicians [19,132]. The usefulness of the CGM would also allow us to assess the effectiveness of therapeutic interventions more accurately [19]. However, these systems are not without their limitations, ranging from low tolerance by some patients to allergic reactions to the devices, as described by a patient with glycogenosis IXb [133].

Historically, the cornerstone of the therapeutic approach has been the prevention of fasting through the implementation of frequent, small meals [6], or, in severe cases, through continuous enteral feeds [134].

The second pillar that has been in place since the 1960s is the introduction of a high-protein diet that also aims to avoid excessive consumption of potentially harmful simple sugars [77]. Proteins use a triple mechanism to be helpful: amino acids are both precursors for gluconeogenesis and direct fuel for muscles, and can reduce glycogen storage by replacing some of the carbohydrates with protein [135]. On this topic, the latest recommendation for GSD IX is the introduction of 2–3 g of protein/kg body weight/day [6].

The first studies focused on finding sources of slow-release carbohydrates that are able to maintain a good blood sugar level for more than 3 h, beginning in the 70 s until uncooked corn starch (UCCS) was established as the most effective therapy [136]. UCCS compared to continuous glucose supplementation has proven to be more beneficial for several reasons: first, maintaining euglycemia requires lower equivalent glucose doses (5.3–7.6 mg/kg/min) compared to those used in continuous feeds (8–10 mg/kg/min), thus lowering the secondary hyperinsulinemia that had been evident in the first years of continuous enteral treatment [137]. A high insulinemia value is, in fact, an additional risk of hypoglycemia if a failure or problem occurs to enteral nutrition continuously, causing a rapid drop in glucose concentrations. UCCS, therefore, has a high neuroprotective impact in the case of hypoglycemia, which is the leading cause of neurological complications [135].

Over the years, numerous reports have been published on the experience and benefits of UCCS, particularly in patients with GSD IXa.

This treatment has sometimes led to the complete resolution of all symptoms [39]. In other, more severe cases of GSD IXa [48], however, the UCCS has led to the resolution of only some symptoms but has not brought benefits to significant symptoms such as stunting [20,38].

Despite being tested in patients before 6 months of age, UCCS is better tolerated beyond 12 months of age due to the immaturity of the digestive enzyme amylase in previous months [135]. One of the valuable indications for evaluating the need to introduce a dose of UCCS in the absence of continuous glucose monitoring may be the monitoring of blood ketones, especially after a night of fasting, since hypoglycemia is not always evident, most notably in GSD IXa [61].

There is also no unambiguous data regarding the dosage, as in the past it was used at very high doses several times a day, while today the indication is usually to give a dose of 1 g/kg before bedtime, in order to maintain a normal blood sugar level for the next 4–8 h (with an average duration of effectiveness of 4.25 h) [6,138].

High doses of UCCS are shown to be both poorly tolerated at the intestinal level and cause increased fat mass and insulinemia, so it is always suggested that patients in this treatment remain constantly under strict nutritional evaluation [41].

In 2008, Glycosade^®^, a waxy, long-release maize, was introduced as an alternative to UCCS, highlighting its ability to extend the duration of fasting (beyond the median 4.25 h of UCCS) and maintain euglycemia.

The most interesting data have recently been published following the Glyde study, the first international randomized double-blind multicenter study [25], comparing Glycosade^®^ and UCCS from 2 years of age (there is no evidence of use before this age).

Regarding fasting tolerance, 100% of the participants tolerated at least 6 h, while 82% tolerated 8 h, thus avoiding the need to awaken in the middle of the night.

Diet and nutrition treatment is effective but demanding and not always well tolerated, e.g., in a recent case report [27] of a GSD IXa with severe liver involvement, the patient could not endure strict dietary control (a diet high in protein and complex carbohydrates) and eventually underwent liver transplantation.

Nowadays, liver transplantation has two main indications: a few selected patients who show relevant symptomatology with poor metabolic control despite reasonable adherence to dietary therapy. The second indication is the presence of hepatic adenomas with malignant transformation, although it should be remembered that the decrease in the levels of triglycerides itself has shown regression of hepatic adenoma [62].

## 4. Discussion

Our systematic review work was primarily based on researching the clinical stigmata and the diagnostic-therapeutic approaches available for the four types of glycogenosis IX described to date.

The literature reviewed includes 400 reported cases, a number that has been steadily increasing in recent years, with 208 cases described in the last 5-year period (2019–2024). To the best of our knowledge, this is the most extensive collection of case histories with glycogenosis IX described in the literature.

Notably, subtype IXa remains the most prevalent form, although its relative proportion has declined slightly (68%), possibly due to increased clinical awareness and improved detection of other subtypes.

The patient profile to be investigated includes those with elevated transaminases or hepatomegaly, especially if in good general health or cases of symptoms such as recurrent episodes of hypoglycemic ketosis, exercise intolerance, or episodic muscle weakness. The first three subtypes (IXa, IXb, and IXc) are typically observed in early childhood, with a mean age at diagnosis of less than 5 years. In contrast, subtype IXd is characteristic of adulthood, with a mean diagnostic age of 44.9 years.

Muscle involvement, classically described mainly in the IXb and IXd forms, is reported almost exclusively in our case series in the IXd form (100% of cases with CPK elevation). In PHKB deficiency, however, it appears to be very rare (only 5.1% of cases), with hepatic involvement being the predominant feature, characterized by hepatomegaly in 79.5% of cases and transaminase elevation in 53.8% of cases.

In X-linked forms, which are by far the most common, the diagnosis should be suspected in the vast majority of cases in male patients, as women are generally asymptomatic carriers, except in homozygotes or cases of X chromosome inactivation patterns [42,88,113].

### 4.1. Hepatic Involvement

Liver involvement is by far the most prominent among the other systems, in all four forms, but especially in GSD IXa, where it is the only symptom in most cases.

In GSD IXa long-term follow-up, various degrees of liver fibrosis (mild, moderate, or severe) were observed in 43.1% of patients, while cirrhosis was reported in a small percentage (8.7%) of cases [35,139].

In the overall population, progression to fibrosis or cirrhosis is uncommon. However, some genotypes, such as PHKG2, may contribute to the development of cirrhosis [24,118]. In GSD IXc, severe hepatomegaly often occurs earlier than in the other subtypes. In one case [30], the co-presence of neonatal jaundice, hepatomegaly, and elevated serum transaminases has been described since the neonatal period. Hepatic adenoma and HCC have been reported exclusively in GSD IXc [49].

### 4.2. Growth

GSD IXa exhibits the highest prevalence of growth retardation (43,4%), which is, however, also much described in the form IXb and IXc, not always associated with short final stature, final data that are unfortunately not always reported in the description of case reports.

Abnormal growth in stature in GSD may be considered as part of an adaptive process whereby growth is suppressed in an attempt to preserve glucose for more essential functions [128]. Unlike GSD types Ia and Ib, where untreated patients are unable to release glucose (and hence experience persistent poor growth), patients with types III and IX show a spontaneous improvement in growth velocity, which may be related to their ability to release glucose via hepatic gluconeogenesis. In fact, as glucose requirements per kilogram of body weight decrease with age, the impact of the enzyme defect lessens over time, reducing its effect on growth. Patients with GSD IX appear to follow a specific growth pattern, characterized by initial growth delay, a late growth spurt, and full catch-up in final height [103].

Another possible speculation is that the presence of chronic ketosis (and consequent acidosis) in younger children inhibits growth, as well as contributes to the development of osteoporosis [135].

After childhood, patients with glycogen storage disease type IX typically exhibit a regular growth pattern, with a distinct clinical improvement from the remaining liver involvement [125].

Another phenomenon to keep in mind is the possibility, in the case where there is simultaneously underlying a true growth hormone (GH) deficiency, of having more severe ketotic hypoglycemias, as reported in a case report [73], for which more treatment may be needed.

Although GSD IX should not be forgotten in the differential diagnosis of short stature in a boy [61], especially with liver involvement, it is more correct to frame this clinical sign as childhood growth retardation, a sign for which an intervention is, therefore, in general not warranted.

### 4.3. Hypoglycemia and Central Nervous System Involvement

Episodes of mild fasting ketotic hypoglycemia seem to be a hallmark of the hepatic forms, more frequent in the more aggressive form (IXc) described in 52.7% of cases. The integrity of the gluconeogenesis pathway may explain why hypoglycemia episodes are usually mild.

The possibility of neurological alteration, especially in GSD IXa, could be either secondary to the episodes of ketotic hypoglycemia or due to the cumulative high incidence of this form of glycogenosis. Patients with neurological manifestations are, in fact, often well-studied subjects, particularly in terms of their genetic profile. Therefore, discovering a variant such as PHKA2 could be an incidental finding that has little to do with clinical manifestation.

The only two reported cases of glycogenosis IXa and autism, given the high prevalence of both conditions, are insufficient to hypothesize an association between them.

In contrast to what has been observed in the past, mild developmental delay has an almost overlapping frequency in form IXc (15.3%) and IXa (14.2%), double that of form IXb.

In the study by Muzetti et al. [42], all patients with IX glycogenosis (five with IXa and one with IXb) underwent brain MRI to compare with other types of glycogenosis. In contrast to other glycogen storage diseases, where brain damage appears to be primarily related to the severity of the glycemic crisis, no significant changes were observed on MRI in any of the patients in this study.

### 4.4. Systemic Involvement

Regarding cardiac involvement, by the late 1990s, five cases of isolated infantile hypertrophic cardiomyopathy of glycogenosis type IX had been collected [109]. The subtype was not specified, as the diagnosis was based on enzyme activity (demonstrated reduced PhK activity) rather than genetics, making this a clinically valuable presentation for the differential diagnosis of infantile hypertrophic cardiomyopathy. In subsequent years, there were no further reports of cardiac involvement, except in one case of glycogen storage disease type IXa [50] and for interventricular septal hypertrophy in 1 case of IXb [84].

Thus, cardiac involvement does not appear to be characteristic of any form of glycogenosis IX; however, it seems reasonable to recommend screening, especially in the muscular forms.

The renal system does not appear to be affected by these forms of glycogenosis, as evidenced by a 2017 study in which all 135 patients with the ketotic forms of GSD (types 0, III, VI, and IX) consistently exhibited normal microalbumin excretion [75].

In 1998, a case of proximal renal tubular acidosis was documented [112] in a patient who appeared to exhibit a therapeutic response to cornstarch treatment. However, no further reports of similar findings have emerged in the subsequent years.

Moreover, some laboratory changes have likely not yet been described because they have not been thoroughly researched or adequately addressed, such as uric acid elevation. In a recent cohort study by Jindan Yu et al. [22], half of the GSD IXa patients had hyperuricemia. The same applies to blood pressure monitoring, as high blood pressure values were also found in one patient [107].

Some endocrinological conditions have also been reported, such as polycystic ovarian syndrome [140].

While the American College of Medical Genetics and Genomics (ACMG) workgroup noted that osteopenia and osteoporosis could potentially occur in GSD IX [6], no documented cases have linked the condition to impaired bone metabolism. In the study of İnci et al. [36], three patients exhibited osteopenia and one showed signs of osteoporosis, despite the absence of hypocalcemia or renal tubulopathy. These findings may be attributed to hyperketosis or lactate levels, as a high level of ketosis is a key indicator of poor metabolic control. Based on the limited amount of available data, it is likely that the deficit in bone mineralization is under-recognized and under-investigated in the follow-up of these patients, an aspect for which there is a need for greater awareness among clinicians.

Some characteristics defined as stigmata, such as the doll-like face, have actually been described only in one patient with GDS IXa, in one with GSD IXb, and in 3 patients with GSD IXc, making this feature quite rare and still not understood [29,112,113].

Therefore, it seems that it is necessary to investigate patients more thoroughly, not limited to hepato-muscular involvement, but also examining endocrinological (especially those related to bone health) and nephrological aspects.

### 4.5. Diagnosis

Diagnosing and distinguishing the hepatic forms of GSD type IX is challenging due to their similar clinical and histological features. In such situations, genetic and molecular analyses have become essential diagnostic tools, according to ACMG guidelines [6] and the latest reports, massively parallel sequencing is the most effective method for identifying and differentiating between the various types of GSD IX.

Advances in massive sequencing have significantly improved genetic diagnostics, enabling the identification of numerous new genomic variants. This has led to a substantial increase in diagnoses, decreased invasiveness and lead time, and lower costs. However, it also presents pitfalls, especially in determining the pathogenicity of the identified variants. Establishing the clinical significance of genetic variants and a robust genotype-phenotype correlation remains challenging, often due to limited access to the affected organ for functional validation [141].

Confidently, emerging studies [17] aimed at refining genotype-phenotype correlations offer promising insights, potentially improving diagnostic accuracy and personalized disease management.

A significant limitation in assessing the pathogenicity of GSD IX remains the lack of reliable data on its true incidence, as the diagnosis is often challenging and usually delayed. It is also evident that a considerable proportion of cases, particularly those with milder symptoms, likely remain undiagnosed. Moreover, this condition is currently excluded from newborn screening due to the absence of a reliable biochemical marker, an issue already encountered in some other metabolic conditions [142].

Considering the low risk of acute metabolic decompensation and the availability of effective supportive interventions to prevent clinical manifestations, glycogenosis type IX constitutes a compelling candidate for inclusion in expanded newborn screening panels [143].

One potential marker under investigation is biotinidase (BTD) serum levels, a test commonly included in newborn screening panels and studied in various types of GSD, including I, III, VI, IX, and XI. However, fluctuations in enzymatic levels, added to intra- and inter-individual variability of the BTD activity in the GSD individuals, led to the conclusion that BTD is not yet a reliable diagnostic biomarker for hepatic GSDs [99,144].

### 4.6. Therapy

In glycogenosis types IXa, IXb, and IXc, the cornerstone of treatment is a personalized and medically prescribed diet, typically including frequent meals, increased protein intake, uncooked cornstarch (UCCS) or Glycosade ^®^, and/or tube feeding, as widely recommended in clinical practice. However, many of the studies reviewed lack specific details regarding the implementation of these strategies—such as the exact dosages, frequency of administration, measurable clinical or laboratory outcomes, and long-term follow-up—often limiting the ability to assess the effectiveness of treatment beyond general statements.

Data from the first randomized double-blind cross-over multicenter international trial were published in 2024, demonstrating the efficacy and tolerance of Glycosade ^®^ in a large cohort of hepatic GSD [25]. The only randomized, double-blind study was published last year, with a follow-up duration of only 2 years.

We definitely need a longer follow-up, as well as more extensive, methodologically sound population studies, to determine which treatment is the most effective.

In contrast, within the limits of the literature included in this review, there is no evidence of therapies and treatment effects in GSD IXd.

There are also no relevant studies on the effect of the ketogenic diet on patients.

### 4.7. Comparison of the Four Phenotypes: Benign or Malignant Condition?

Based on the systematic review of reported cases to date, individuals with GSD IXc typically exhibit a more severe clinical phenotype and a more significant potential for aggressive disease progression compared to GSD IXa, IXb, and IXd.

PHKG2-related liver phosphorylase kinase deficiency can, in fact, exhibit phenotypic features that resemble both glycogen storage disease types I and III [52]. This observation can be substantiated at the molecular level by considering that the γ subunit, which harbors the enzyme’s catalytic site, is essential for its function. Disruption of this specific region, rather than the regulatory domains, is likely to cause a more profound functional impairment of the enzyme, thereby leading to more severe disease manifestations [17].

With this perspective, a more detailed investigation into the functional roles of the different subunits and their impact on enzymatic activity could yield valuable insights into the disease mechanism and inform the development of targeted therapeutic strategies aimed at these critical sites of disruption.

GSD IXd is a relatively recently characterized entity that typically follows a benign course and may even be asymptomatic or present as an adult-onset distal calf-dominant myopathy, which does not always manifest with exercise intolerance. However, this pattern is not universally observed. Notably, the case reported by Picillo et al. describes a more severe muscle phenotype without cardiac or respiratory involvement. This atypical presentation may be attributed to compound heterozygous variants in the GAA gene, inherited from both parents, underscoring the potential impact of genetic variability on disease expression.

A notable finding of our systematic review is the substantial rise in the number of diagnoses over the past decade, with a particularly marked increase observed in the last 5 years.

In addition to these data, which are easily justifiable given the considerable progress made in genetic panels, their increased availability, and lower overall costs, we have also observed a progressive reduction in the severity of symptoms in diagnosed patients.

The decrease in the clinical severity of cases described in recent years (in which symptoms such as motor/cognitive retardation or advanced liver fibrosis are rarely reported) is, however, likely due precisely to the greater genetic diagnostic capacity available and the increased attention of clinicians to early diagnosis.

A fascinating case history is described in some Danish families [47] in which patients presented with ketotic hypoglycemia only, without transaminase elevation or hepatomegaly. In adulthood, patients who shared the same mutations had some persistence of ketotic hypoglycemia, while others became asymptomatic.

Interestingly, in a recent Italian large cohort [145], only minimal changes were observed during follow-up in GSD IX patients.

In response to the work of Fernandes et al. [139], which questioned the benign nature of the condition, we believe that, despite its considerable phenotypic variability, it should not be overlooked, as it warrants clinical attention and early diagnosis.

Evaluating the condition’s progress with long-term follow-up will be interesting, as it may improve in adulthood.

The most favorable perspective is that, with a positive response to the therapeutic approach, early diagnosis and timely treatment will soon make this condition as asymptomatic as possible.

This systematic review has several limitations. First, the data were derived from case reports and case series, which are inherently subject to selection and publication biases. The heterogeneity in reporting clinical features, laboratory findings, and genetic data limited the ability to perform a quantitative synthesis or formal statistical comparisons. In many cases, essential clinical or biochemical parameters were missing or inconsistently reported, preventing a more robust stratification of results. Additionally, no standardized tool was used to assess the quality or risk of bias in the included studies. In most cases, the absence of long-term follow-up data also limits our understanding of the natural history and long-term complications of GSD IX. Finally, as no formal meta-analysis was conducted, the findings should be interpreted with caution and considered primarily descriptive.

## 5. Conclusions

This systematic review includes the most extensive clinical case history of GSD IX described in the literature. The significant increase in diagnoses over the last 5 years, mainly due to significant advances in the field of genetics, makes us look at this disease as an emerging issue of considerable interest in the field of rare diseases. A child who clinically presents some characteristic red flags in the first 5 years of life, primarily liver involvement and ketotic hypoglycemia, alone or associated with the other symptoms described in the review, should be investigated for these genes as soon as possible. This disease also appears to be an ideal candidate for inclusion in a neonatal screening project, with the possibility to secure the newborn with some dietary or therapeutic precautions that can prevent the not-uncommon sequelae. The multiplicity of clinical symptoms and even severe liver involvement make us look at this condition as a malignant phenotype, or certainly one worthy of diagnosis and monitoring.

Finally, throughout the review process, we identified three key areas that remain underexplored and represent potential targets for future research, where robust evidence is currently lacking:

1. the absence of well-designed studies on the long-term outcomes of this patient cohort;
2. the need for deeper insights into genotype-phenotype correlations; and
3. the lack of detailed comparative studies evaluating the impact of dietary and therapeutic management.

## Figures and Tables

**Table 1:** Classification of pathogenetic GSD type IX.

Pathology	Genes	Inheritance ^1^	Enzyme Subunit	Function	Affected Tissue
GSD IXa	PHKA2	XL	α	Regulatory	Liver
GSD IXb	PHKB	AR	β	Regulatory	Muscle; liver
GSD IXc	PHKG2	AR	γ	Catalytic	Liver
GSD IXd	PHKA1	XL	α	Regulatory	Muscle

^1^ Inheritance: autosomal recessive (AR), X-linked (XL).

**Table 2:** Research results with key clinical and laboratory characteristics of patients.

	GSD IXa Number of Patients (%)	GSD IXb Number of Patients (%)	GSD IXc Number of Patients (%)	GSD IXd Number of Patients (%)
**Total**	274	39	72	15
**Female/male**	8 (8.8%)/83 (91.2%)	7 (35%)/13 (65%)	16 (45.7%)/19 (54.3%)	1 (6.7%)/14 (93.3%)
**Mean age at onset (years)**	/	/	/	35.9
**Mean age at diagnosis (years)**	5.1	4.9	3.2	44.9
**Number of liver biopsies**	82 (29.9%)	10 (25.6%)	41 (56.9%)	11 muscle (73.3%); 1 liver + muscle (6.7%)
**Mean age at liver biopsy (years)**	5.1	/	4.8	48.3 (muscle biopsy)
**Hepatomegaly**	205 (74.8%)	31 (79.5%)	66 (91.7%)	1 (6.7%)
**Fasting hypoglycemia**	79 (28.8%)	10 (25.6%)	38 (52.7%)	0 (0%)
**Fasting ketosis**	43 (15.7%)	3 (7.7%)	8 (11.1%)	1 (6.7%)
**Elevated ALT/AST**	175 (63.9%)	21 (53.8%)	58 (80.6%)	1 (6.7%)
**Mean AST (U/L)**	392 (n = 51, range 24–2067)	278 (n = 9, range 74–660)	513 (n = 10, range 42–1006)	/
**Mean ALT (U/L)**	270 (n = 72, range 23–1299)	194 (n = 12, range 50–600)	379 (n = 28, range 67–1235)	/
**Hypertriglyceridemia**	96 (35.0%)	15 (38.4%)	37 (51.4%)	0 (0%)
**Hypercholesterolemia**	55 (20.1%)	5 (12.8%)	8 (11.1%)	0 (0%)
**Growth delay/short stature**	120 (43.8%)	15 (38.4%)	30 (41.7%)	1 (6.7%)
**Developmental delay**	39 (14.2%)	3 (7.7%)	11 (15.3%)	0 (0%)
**Muscle involvement**	6 (2.2%)	2 (5.1%)	0 (0%)	14 (93.3%)
**Elevated CPK**	5 (1.8%)	2 (5.1%)	0 (0%)	15 (100%)
**Mean CPK (U/L)**	/	331 (n = 3, range 88–562)	88 (n = 13, range 46–141)	1011 (n = 14, range 120–2842)
**Neurological alterations**	1 neurosensorial hearing loss, 2 seizures, 2 autism, 2 cerebellar involvement	1 neonatal hypoglycemic coma	14 cases of seizures (19.4%)	2 cases of cognitive impairment
**Hepatic adenoma**	1 (0.4%)	1 (5.6%)	5 (6.7%)	0 (0%)
**Liver transplant**	1 (0.4%)	0 (0%)	1 (1.4%)	0 (0%)
**Other symptoms**	2 renal tubular acidosis, 1 lactic acidosis, 1 osteoporosis, 1 anemia, 7 multiple infections, 1 Acute pancreatitis, 2 chronic diarrhea, 1 doll-like face, 1 hypertrophic cardiomyopathy	1 doll-like face, 1 low WBC, 1 interventricular septal hypertrophy	3 doll-like faces	/

## Data Availability

All clinical data and materials are available in our Pediatric Unit.

## Supplementary Materials

The following supporting information can be downloaded at: https://www.mdpi.com/article/10.3390/genes16050584/s1, Table S1. PRISMA 2020 statement: an updated guideline for reporting systematic reviews; Table S2. Collection of selected works. CR: case report. CS: case series. RCT: randomized controlled trial.

## Abbreviations

The following abbreviations are used in this manuscript:

GSD	Glycogen storage disease
PhK	Phosphorylase kinase
PYGL	Liver glycogen phosphorylase
AR	Autosomal recessive
XL	X-linked
CGM	Continuous glucose monitoring
UCCS	Uncooked corn starch
