# Supplementary material for: Understanding Glycogen Storage Disease Type IX: A Systematic Review with Clinical Focus—Why It Is Not Benign and Requires Vigilance
Source: Genes (Basel). 2025 May 15;16(5):584. doi: 10.3390/genes16050584 (PMC12111550; doi:10.3390/genes16050584)
Supplement: Supplementary file 1 [file genes-16-00584-s001.zip › Suppl. Table S2.pdf]

Title & Authors	Year	Country	Study type	n. GSD IX patients (subtype)
**A rare co-occurrence of phosphorylase kinase deficiency (GSD type IXd) and alpha-glycosidase deficiency (GSD Type II) in a 53-year-old man presenting with an atypical glycogen storage disease phenotype** Picillo E. et al.	2024	Italy	**CR**	1 (IXd)
**Report of an Iranian child with chronic abdominal pain and constipation diagnosed as glycogen storage disease type IX: a CR** Zamanfar, D. et al.	2024	Iran	**CR**	1 (IXb)
**Continuous glucose monitoring metrics in people with liver glycogen storage disease and idiopathic ketotic hypoglycemia: A single-center, retrospective, observational study** Overduin R.J. et al.	2024	Netherlands, Italy	**CS**	9 (unknown)
**Molecular profiles and long-term outcomes of Thai children with hepatic glycogen storage disease in Thailand** Vanduangden J. et al.	2024	Thailand	**CS**	1 (IXa)
**Broadening the Phenotype and Genotype Spectrum of Glycogen Storage Disease by Unraveling Novel Variants in an Iranian Patient Cohort** Moghimi P. et al.	2024	Iran	**CS**	I (IXa)
**Genotypic and phenotypic features of 39 Chinese patients with glycogen storage diseases type I, VI, and IX** Yu J et al.	2024	China	**CS**	15 (12 IXa, 3 IXb)
**Whole-exome sequencing for genetic diagnosis of idiopathic liver injury in children.** Lülecioğlu AA et al.	2024	Turkey	**CR**	1 (IXa)
**Asymptomatic cirrhosis in a 35-year-old female** Yao X. et al.	2024	China	**CR**	1 (IXc)
**Short and long-term acceptability and efficacy of extended-release cornstarch in the hepatic glycogen storage diseases: results from the Glyde study** Weinstein D.A. et al.	2024	USA, UK	**RCT**	14 (unknown)
**Identification of a novel deletion mutation in PHKA2 in a taiwanese patient with type IXa glycogen storage disease** Lin WD et al.	2023	Taiwan	**CR**	1 (IXa)
**A novel PHKA2 variant in a Chinese boy with glycogen storage diseases type IXa.** Zhu H. et al.	2023	China	**CR**	1 (IXa)
**Glycogen Storage Disease: Expert Opinion on Clinical Diagnosis Revisited after Molecular Testing** De Marchi R. et al.	2023	Brazil	**CS**	13 (6 IXa + 4 IXb + 3 IXc)
**Clinical, pathological and molecular spectrum of patients with glycogen storage diseases in Pakistan** Ahmed S. et al.	2022	Pakistan	**CS**	9 (IXc 6 + IXb 2 + IXa 1)
**A very rare case report of glycogen storage disease type IXc with novel PHKG2 variants** Shao Y. et al.	2022	China	**CR**	1 (IXc)
**Glycogen Storage Disease Type IXb in a Neonate Caused by Two Mutations in PHKB Gene** Arumugam S.K. et al.	2022	India	**CR**	1 (IXb)
**A 78-year-old Japanese male with late-onset PHKA1-associated distal myopathy: Case report and literature review** Mori-Yoshimura M. et al.	2022	Japan	**CR**	1 (IXd)
**Whole-exome Sequencing Analysis of a Japanese Patient With Hyperinsulinemia and Liver Dysfunction**	2022	Japan	**CR**	1 (unknown)

Fujita S. et al.				
**Modeling a Novel Variant of Glycogenosis IXa Using a Clonal Inducible Reprogramming System to Generate “Diseased” Hepatocytes for Accurate Diagnosis**<br>Garcia-Llorens G. et al.	2022	Spain	**CR**	1 (IXa)
**Identification of a novel mutation in the PHKA2 gene in a child with liver cirrhosis**<br>Beyzaei Z. et al.	2022	Iran, Turkey	**CR**	1 (IXa)
**Expected or unexpected clinical findings in liver glycogen storage disease type IX: distinct clinical and molecular variability**<br>İnci A. et al.	2022	Turkey	**CS**	25 (16 IXa, 6 IXc, 3 IXb)
**Molecular and clinical profiling in a large cohort of Asian Indians with glycogen storage disorders**<br>Kumar T.V. et al.	2022	India	**CS**	11 (2 IXa, 3 IXb, 6 IXc)
**Genotypic and phenotypic characteristics of 12 chinese children with glycogen storage diseases**<br>Dong R. et al.	2022	China	**CR**	2 (IXa)
**A novel PHKA2 variant in a Japanese boy with glycogen storage diseases type IXa**<br>Mori T. et al.	2022	Japan	**CR**	1 (IXa)
**Maximal Multistage Shuttle Run Test-induced Myalgia in a Patient with Muscle Phosphorylase B Kinase Deficiency**<br>Munekane A. et al.	2022	Japan	**CR**	1 (IXd)
**Body composition in patients with hepatic glycogen storage diseases**<br>dos Santos B.B. et al.	2022	Brazil	**CS**	4 (IXa, IXb, IXc)
**Neurological Characteristics of Pediatric Glycogen Storage Disease**<br>Muzetti J.H. et al.	2021	Brazil	**CS**	6 (5 IXa, 1 IXb)
**Novel mutations in the PHKB gene in an iranian girl with severe liver involvement and glycogen storage disease type IX: a CR and review of literature**<br>Beyzaei Z. et al.	2021	Iran	**CR**	1 (IXb)
**Clinical and genetic spectrum of glycogen storage disease in Iranian population using targeted gene sequencing**<br>Beyzaei Z. et al.	2021	Iran	**CR**	3 (2 IXb, 1 IXc)
**Profound neonatal lactic acidosis and renal tubulopathy in a patient with glycogen storage disease type IXα2 secondary to a de novo pathogenic variant in PHKA2**<br>Morales J.A. et al.	2021	USA	**CR**	1 (IXa)
**Autism: Screening of inborn errors of metabolism and unexpected results**<br>İnci A. et al.	2021	Turkey	**CR**	1 (IXa)
**PHKA2 variants expand the phenotype of phosphorylase B kinase deficiency to include patients with ketotic hypoglycemia only**<br>Benner A. et al.	2021	Denmark	**CS**	16 (IXa)
**Male inheritance of X-linked liver glycogenosis from an undiagnosed maternal grandfather in a Chinese pedigree: a report of two cases**<br>Li P. et al.	2021	China	**CR**	2 (IXa)
**A female patient with GSD IXc developing multiple and recurrent hepatocellular carcinoma: a case report and literature review**<br>Kido J. et al.	2021	Japan	**CR**	1 (IXc)
**Evaluation of glycogen storage patients: Report of twelve novel variants and new clinical findings in a Turkish population**<br>Ersoy M. et al.	2021	Turkey	**CS**	6 (4 IXa + 1 IXb + 1 IXd)

**A novel PHKA1 mutation associating myopathy and cognitive impairment: Expanding the spectrum of phosphorylase kinase b (PhK) deficiency**<br>Bisciglia M. et al.	2021	Belgium	**CR**	2 (siblings) (IXd)
**Variability of clinical and biochemical phenotype in liver phosphorylase kinase deficiency with variants in the phosphorylase kinase (PHKG2) gene**<br>Waheed N. et al.	2020	Pakistan	**CS**	10 (IXc)
**An unusual case of recurrent episodes of muscle weakness: Co-occurrence of Andersen-Tawil syndrome and glycogen storage disease type IXd**<br>Li H. et al.	2020	China	**CR**	1 (IXd)
**Liver histology in children with glycogen storage disorders type VI and IX**<br>Degrassi I. et al.	2020	UK, Italy	**CS**	13 (9 IXa, 1 IXb and 3 IXc)
**Molecular diagnosis of glycogen storage disease type IX using a glycogen storage disease gene panel**<br>Kim T.H. et al.	2020	South Korea	**CS**	10 (8 IXa, 2 IXc)
**Over 20-year follow-up of patients with hepatic glycogen storage diseases: Single-center experience**<br>Szymańska E. et al.	2020	Poland	**CR**	1 (IXa)
**A novel frameshift PHKA2 mutation in a family with glycogen storage disease type IXa: A first report in Vietnam and review of literature**<br>Nguyen N.-L. et al.	2020	Vietnam	**CR**	2 (IXa)
**Genotypic and clinical analysis of 49 Chinese children with hepatic glycogen storage diseases**<br>Liang Y. et al.	2020	China	**CS**	8 (IXa)
**Neurological Involvement in Glycogen Storage Disease Type IXa due to PHKA2 Mutation**<br>Smith C. et al.	2020	Canada	**CR**	2 (IXa)
**No effect of oral sucrose or IV glucose during exercise in phosphorylase b kinase deficiency**<br>Andersen A.G. et al.	2020	Denmark	**CR**	1 (IXd)
**Glycogen storage disease IXa in a 9-year-old Filipino boy with short stature: A case report**<br>Estrada S.C. et al.	2020	Philippines	**CR**	1 (IXa)
**Hepatic Glycogenoses Among Children—Clinical and Biochemical Characterization: Single-Center Study**<br>Korula S. et al.	2020	India	**CR**	2 (1 IXa + 1 IXc)
**Mutation in PHKA2 leading to childhood glycogen storage disease type IXa: A CR and literature review**<br>Zhu Q. et al.	2019	China	**CR**	1 (IXa)
**Quality of life as a criterion for assessing the health status of children with glycogen storage diseases**<br>Surkov A.N. et al.	2019	Russia	**CS**	13 (IX and VI)
**A novel PHKA2 mutation in a Chinese child with glycogen storage disease type IXa: A CR and literature review**<br>Fu J. et al.	2019	China	**CR**	1 (IXa)
**Fatty Liver Caused by Glycogen Storage Disease Type IX: A Small Series of Cases in Children**<br>Leuzinger Dias C. et al.	2019	Portugal	**CR**	3 (IXa)
**PHKG2 mutation spectrum in glycogen storage disease type IXc: A CR and review of the literature**<br>Li C et al.	2019	China	**CR**	1 (IXc)
**Clinical and genetic characteristics of three Chinese patients with glycogen storage disease type IXa**<br>Yang F. et al.	2019	China	**CS**	3 (IXa)
**Role of continuous glucose monitoring in the management of glycogen storage disorders**<br>Herbert M. et al.	2018	USA	**CS**	5 (1 IXa, 2 IXb, 2 IXc)
**Aberrant apolipoprotein C-III glycosylation in glycogen storage disease type III and IX**	2018	Czech Republic, Slovakia	**CS**	5 (unknown)

Ondruskova N. et al.				
**A new variant in PHKA2 is associated with glycogen storage disease type IXa**<br>Rodríguez-Jiménez C et al.	2017	Spain	**CR**	1 (IXa)
**Clinical and molecular variability in patients with PHKA2 variants and liver phosphorylase b kinase Deficiency**<br>Bali D.S. et al.	2017	USA	**CS**	12 (IXa)
**Glycogen storage disease type IX and growth hormone deficiency presenting as severe ketotic hypoglycemia**<br>Hodax J.K. et al.	2017	USA	**CR**	1 (IXa)
**Clinical and genetic characteristics of 17 Chinese patients with glycogen storage disease type IXa**<br>Zhang J. et al.	2017	China	**CS**	17 (IXa)
**Tight metabolic control plus ACE inhibitor therapy improves GSD I nephropathy**<br>Okechuku G.O. et al.	2017	USA	**CS**	58 (unknown)
**PHKA2 mutation spectrum in Korean patients with glycogen storage disease type IX: Prevalence of deletion mutations**<br>Choi R. et al.	2016	Korea	**CS**	6 (IXa)
**Dietary management of the ketogenic glycogen storage diseases**<br>Bhattacharya K. et al.	2016	Australia	**CR**	2(IXc)
**Structured dietary management dramatically improves marked transaminitis, metabolic and clinical profiles in glycogen storage disease type IXa**<br>Karande I.S. et al.	2016	Australia	**CR**	1 (IXa)
**Detection and Quantification of Mosaic Mutations in Disease Genes by Next-Generation Sequencing**<br>Qin L. et al.	2016	USA	**CR**	I (IXa)
**Clinical, biochemical, and genetic characterization of glycogen storage type IX in a child with asymptomatic hepatomegaly**<br>Kim J.A. et al.	2015	South Korea, Germany	**CR**	1 (unknown)
**Evaluation of glycogen storage disease as a cause of ketotic hypoglycemia in children**<br>Brown L.M. et al.	2015	USA	**CS**	14 (12 IXa, 1 IXb, 1 IXc)
**Variability of disease spectrum in children with liver phosphorylase kinase deficiency caused by mutations in the PHKG2 gene**<br>Bali DS et al.	2014	USA, Australia	**CS**	5 (IXc)
**Novel PHKG2 mutation causing GSD IX with prominent liver disease: Report of three cases and review of literature**<br>Albash B. et al.	2014	Saudi Arabia, Kuwait	**CR**	3 (IXc)
**Whole exome sequencing unravels disease-causing genes in consanguineous families in Qatar**<br>Fahiminiya S. et al.	2014	Qatar	**CR**	1 (IXc)
**The natural history of glycogen storage disease types VI and IX: Long-term outcome from the largest metabolic center in Canada**<br>Roscher A. et al.	2014	Canada	**CS**	17 (11 IXa, 3 IXb, 3 IXc)
**Bariatric surgery is not contraindicated in obese patients suffering from glycogen storage disease type IXa. A case report with follow-up at three years**<br>Musella M. et al**.**	2014	Italy	**CR**	1 (IXa)
**Aggressive therapy improves cirrhosis in glycogen storage disease type IX**<br>Tsilianidis L.A. et al.	2013	USA	**CR**	2 (IXa)
**Clinical application of massively parallel sequencing in the molecular diagnosis of glycogen storage diseases of genetically heterogeneous origin**	2013	USA	**CS**	4 (3 IXa, 1 IXc)

Wang J. et al.				
**X-linked glycogen storage disease IXa manifested in a female carrier due to skewed X chromosome inactivation** Cho S.Y. et al.	2013	China	**CR**	1 (IXa)
**Glycogen storage disease type IX: Novel PHKA2 missense mutation and cirrhosis** Johnson A.O. et al.	2012	USA	**CR**	1 (IXa)
**Muscle phosphorylase kinase deficiency: a neutral metabolic variant or a disease?** Preisler N. et al.	2012	Denmark	**CR**	2 (IXd)
**Novel mutations in PHKA2 gene in glycogen storage disease type IX patients from Hong Kong, China** Lau C.-K. et al.	2011	China	**CR**	1 (IXa)
**Common mutation in the PHKA2 gene with variable phenotype in patients with liver phosphorylase b kinase deficiency** Achouitar S. et al.	2011	USA, Netherlands	**CS**	14 (IXa)
**Liver glycogen storage diseases due to phosphorylase system deficiencies: Diagnosis thanks to non invasive blood enzymatic and molecular studies** Davit-Spraul A. et al.	2011	France	**CS**	32 (26 IXa, 3 IXb, 3 IXc)
**A novel PHKA2 gross deletion mutation in a Korean patient with X-linked liver glycogenosis type I** Park K.-J. et al.	2011	Korea	**CR**	1 (IXa)
**Muscle phosphorylase b kinase deficiency revisited** Echaniz-Laguna A. et al.	2010	France	**CR**	1 (IXd)
**Evaluation of the biotinidase activity in hepatic glycogen storage disease patients. Undescribed genetic finding associated with atypical enzymatic behavior: an outlook** Angaroni CJ et al.	2010	Argentina	**CS**	5 (unknown)
**X-linked Liver Glycogenosis in a Taiwanese Family: Transmission From Undiagnosed Males** Chen S.-T. et al.	2009	Taiwan	**CR**	2 (IXa)
**Is muscle glycogenolysis impaired in X-linked phosphorylase b kinase deficiency?** Ørngreen M.C. et al.	2008	Denmark	**CR**	1 (IXd)
**Identification of Alu-mediated, large deletion-spanning introns 19-26 in PHKA2 in a patient with X-linked liver glycogenosis (hepatic phosphorylase kinase deficiency)** Fukao T. et al.	2007	Japan	**CR**	1 (IXa)
**Elevated serum biotinidase activity in hepatic glycogen storage disorders - A convenient biomarker** Paesold-Burda P. et al.	2007	Switzerland, Germany	**CS**	22 (unknown)
**Glycogen storage disease type IX: High variability in clinical phenotype** Beauchamp N.J. et al.	2007	UK	**CS**	16 (11 IXa, 3 IXb, 2 IXc)
**Multiple voxel 1H MR spectroscopy of phosphorylase-b kinase deficient patients (GSD IXa) showing an accumulation of fat in the liver that resolves with aging** Sijens P.E. et al.	2006	Netherlands	**CR**	8 (IXa)
**Myopathy and phosphorylase kinase deficiency caused by a mutation in the PHKA1 gene** Wuyts W. et al.	2005	Belgium	**CR**	1 (IXd)
**A novel mutation of the PHKA2 gene in a patient with X-linked liver glycogenosis type 1** Hidaka F. et al.	2005	Japan	**CR**	1 (IXa)
**Characteristic growth pattern in male X-linked phosphorylase-b kinase deficiency (GSD IX)** Schippers H.M. et al.	2003	Netherlands	**CS**	51 (IXa)

**Detection of PHKA2 gene mutation in four Japanese patients with hepatic phosphorylase kinase deficiency**<br>Ban K. et al.	2003	Japan	**CS**	4 (IXa)
**Muscle glycogenosis with low phosphorylase kinase activity: Mutations in PHKA1, PHKG1 or six other candidate genes explain only a minority of cases**<br>Burwinkel B. et al.	2003	Germany	**CS**	6 (undetermined)
**Severe Phenotype of Phosphorylase Kinase-Deficient Liver Glycogenosis with Mutations in the PHKG2 Gene**<br>Burwinkel B. et al.	2003	Canada	**CR**	3 (IXa)
**Circadian pattern of blood pressure, heart rate, and double product in liver glycogen storage disease**<br>Yetman R.J. et al.	2002	USA	**CS**	2 (unknown)
**Identification of three novel mutations in the PHKA2 gene in Czech patients with X-linked liver glycogenosis**<br>Rudolfová J. et al.	2001	Czech Republic	**CS**	4 (IXa)
**Infantile hypertrophic cardiomyopathy of glycogenosis type IX: Isolated cardiac phosphorylase kinase deficiency**<br>Regalado J.J. et al.	1999	USA	**CR**	2 (unknown)
**Complete genomic structure and mutational spectrum of PHKA2 in patients with X-linked liver glycogenosis type I and II**<br>Hendrickx J. et al.	1999	UK, USA, France, Belgium	**CS**	10 (IXa)
**Mutational analyses in four Japanese families with X-linked liver phosphorylase kinase deficiency type 1**<br>Hirono H. et al.	1998	Japan	**CS**	6 (IXa)
**Variability of biochemical and clinical phenotype in X-linked liver glycogenosis with mutations in the phosphorylase kinase PHKA2 gene**<br>Burwinkel B. et al.	1998	England	**CS**	4 (IXa)
**Clinical, biochemical and molecular findings in a patient with X-linked liver glycogenosis followed for 40 years**<br>Hendrickx J. et al.	1998	Belgium, Switzerland	**CR**	1 (IXa)
**Autosomal glycogenosis of liver and muscle due to phosphorylase kinase deficiency is caused by mutations in the phosphorylase kinase β subunit (PHKB)**<br>Burwinkel B. et al.	1997	Germany	**CS**	5 (IXb)
**Autosomal recessive liver phosphorylase kinase deficiency caused by a novel splice-site mutation in the gene encoding the liver gamma subunit (PHKG2)**<br>Van Beurden E.A. et al.	1997	The Netherlands	**CR**	2 (IXc)
**Phosphorylase-kinase-deficient liver glycogenosis with an unusual biochemical phenotype in blood cells associated with a missense mutation in the β subunit gene (PHKB)**<br>Burwinkel B. et al.	1997	Germany	**CR**	1 (IXb)
**X-linked liver glycogenosis type II (XLG II) is caused by mutations in PHKA2, the gene encoding the liver α subunit of phosphorylase kinase**<br>Hendrickx J. et al.	1996	Belgium, Germany, Netherland	**CS**	12 (IXa)
**Mutation hotspots in the PHKA2 gene in X-linked liver glycogenosis due to phosphorylase kinase deficiency with atypical activity in blood cells (XLG2)**<br>Burwinkel B. et al.	1996	Germany, Netherland,Austria, France	**CS**	5 (IXa)
**Mutations in the testis/liver isoform of the phosphorylase kinase gamma subunit (PHKG2) cause autosomal liver glycogenosis in the gsd rat and in humans**<br>Maichele A.J. et al.	1996	Germany, France, Norway	**CR**	3 (IXc)

**Phosphorylase b kinase deficiency glycogenosis with cirrhosis of the liver**<br>Kagalwalla A.F. et al.	1995	Saudi Arabia	**CR**	1 (unknown)
**Glomerular and tubular function in glycogen storage disease**<br>Lee P.J. et al.	1995	UK	**CS**	22 (unknown)
**Mutations in the phosphorylase kinase gene PHKA2 are responsible for X-linked liver glycogen storage disease**<br>Hendrickx J. et al.	1995	England, France, Belgium	**CS**	12 (IXa)
**Isolation of cDNA encoding the human liver phosphorylase kinase α subunit (PHKA2) and identification of a missense mutation of the PHKA2 gene in a family with liver phosphorylase kinase deficiency**<br>Hirono H. et al.	1995	Japan	**CR**	I (IXa)
**Localization of a new type of X-linked liver glycogenosis to the chromosomal region Xp22 containing the liver α-subunit of phosphorylase kinase (PHKA2)**<br>Hendrickx J. et al.	1994	Belgium, France, Netherland, Germany, United Kingdom	**CS**	24 (IXa)
**X-linked liver glycogenosis: Localization and isolation of a candidate gene**<br>Hendrickx J. et al	1993	Belgium, France, Netherland, Germany, United Kingdom	**CS**	20 (IXa)
**Biochemical diagnosis of hepatic glycogen storage diseases: 20 years French experience**<br>Maire I. et al.	1991	France	**CS**	61 (unknown)
**Adult phosphorylase b kinase deficiency**<br>Clemens P.R. et al.	1990	USA	**CR**	2 (unknown)
**The long-term outcome of patients with glycogen storage diseases**<br>Smit G.P.A. et al.	1990	Netherland, UK, Israel	**CS**	43 (VI + IX)
**Phosphorylase kinase in leukocytes and erythrocytes of a patient with glycogen storage disease type IX**<br>Bashan N. et al.	1987	Israele	**CR**	1 (unknown)
**A New Variant of Glycogen Storage Disease: Type IXc**<br>Lerner A. et al.	1982	Israel	**CR**	3 (IXc)
**Growth and endocrine changes in the hepatic glycogenoses**<br>Dunger D.B. et al.	1982	UK	**CR**	2 (unknown)

Supplementary Table S2. Collection of selected works. CR: Case Report. CS: Case Series. RCT: Randomized Controlled Trial.
